# Functional genomic profiling of schizophrenia-associated genes reveals key microglial regulators

**DOI:** 10.1038/s41386-026-02406-1

**Published:** 2026-04-16

**Authors:** Joy E. Horng, Liam T. McCrea, Rebecca E. Batorsky, Joshua J. Bowen, Camilla Boschian, Yoonjae Song, Roy H. Perlis, Steven D. Sheridan

**Affiliations:** 1 Center for Genomic Medicine and Department of Psychiatry, Massachusetts General Hospital, Boston, MA USA; 2 Department of Molecular Biology, Massachusetts General Hospital, Boston, MA USA; 3 Tufts Institute for Artificial Intelligence, Tufts University, Medford, MA USA; 4 Department of Psychiatry, Harvard Medical School, Boston, MA USA; 5 Harvard Stem Cell Institute, Cambridge, MA USA

**Keywords:** Cellular neuroscience, Genetics of the nervous system

## Abstract

Microglia are increasingly recognized as key regulators of neural circuit development and putative contributors to the pathophysiology of neuropsychiatric disorders such as schizophrenia (SCZ). However, the functional impact of SCZ-associated genes in microglia remains largely unexplored. Here, we performed an arrayed CRISPR targeting screen of 30 SCZ-associated genes predicted to be differentially expressed in human microglia-like cells. Target genes were prioritized based on post-mortem transcriptomic relevance and predicted ontology-based roles in phagocytosis pathways. We quantified phagocytic activity and morphological changes following gene targeting using high-content confocal imaging. Key targets, including *CYFIP1, MSR1, TREM2, SYK, ITGB2*, *ITGAM*, and *IRF8*, modulated phagocytosis and altered morphological properties consistent with activation states, validating their functional roles in microglia. To elucidate transcriptional impact, we further applied a multiplexed RNA sequencing platform across gene targets. These analyses revealed gene-specific transcriptional signatures, implicating divergent pathways related to phagocytic, activation, cytoskeletal, and lysosomal function. Together, these findings demonstrate the utility of CRISPR-based functional genomics in characterizing microglia function and identifying new target genes and mechanisms that may underlie their contributions to SCZ pathophysiology.

## Introduction

Schizophrenia (SCZ) is a genetically complex, highly heritable neuropsychiatric disorder characterized by symptoms including psychosis, cognitive deficits, and social withdrawal, typically emerging in late adolescence or early adulthood [1, 2]. SCZ is highly polygenic [3]: Genome-wide association studies (GWAS) have identified hundreds of SCZ-associated loci, including several within immune-related genes [4–6], suggesting an overlap between synaptic and neuroimmune pathways [4, 5]. Convergent evidence for neuroimmune mechanisms comes from large-scale postmortem RNA sequencing studies [7], suggesting some risk genes are highly expressed in microglia [8, 9].

These brain-resident immune cells are key regulators of neural development and function [10, 11]. They mediate synaptic pruning via complement-dependent and less understood mechanisms to maintain homeostasis through surveillance and phagocytosis [12–15]. Postmortem, live imaging, and transcriptomic studies implicate increased inflammatory gene expression, and altered microglial morphology and activity in SCZ [16–20]. Further, patient-derived in vitro models demonstrate excessive microglial synaptic engulfment in microglia-like cells derived from individuals with SCZ [21], yet the functional consequences of disrupting SCZ-associated genes in human microglia remain unknown.

To facilitate the investigation of human microglia, multiple in vitro cellular models have been developed using either induced pluripotent stem cell (iPSC) differentiation [22–24] or direct transdifferentiation from peripheral blood mononuclear cells (PBMCs) [21, 25, 26], which permit higher throughput screening [27] of disease-relevant phenotypes. CRISPR-based platforms, such as pooled screens of the druggable genome in Cas9-expressing engineered iPSC-derived microglia-like cells, have identified genes involved in survival and function [28]. However, despite extensive CRISPR-based functional genomics investigation in neurons [29–31], systematic in vitro genetic screens targeting SCZ-risk genes in microglia remain scarce.

We leveraged an arrayed CRISPR targeting approach in PBMC-derived human microglia-like cells (piMGLCs) to investigate the functional roles of SCZ-associated genes. Target genes were identified based on postmortem differentially expressed genes (DEGs) and expression in human microglia, with prioritization using predicted involvement in phagocytosis-related pathways from gene enrichment analyses. We first conducted primary functional screening, which assessed piMGLC phagocytosis of human synaptosomes in an immunocytochemistry and high-content confocal imaging-based in vitro model of synaptic pruning [21, 25–27]. Genes that altered phagocytosis upon gene targeting were selected for orthogonal secondary screening using redesigned sgRNAs to validate their effects on phagocytic activity and measure indicators of activation state, including cellular morphology and marker expression. Finally, we employed a high-throughput RNA sequencing platform [32] to profile the transcriptional impact across gene targets, enabling the discovery of downstream pathways and potential mechanisms of action. We hypothesized that specific SCZ-associated genes would modulate microglial activity, thereby contributing to the synaptic alterations observed in postmortem and in vitro studies. Beyond offering novel insights into microglia-mediated mechanisms in SCZ, this approach establishes a scalable platform for neuroimmune genetic screening.

## Methods and Materials

### Ethical statement

The study was approved by the Mass General Brigham Institutional Review Board (IRB). Informed consent was obtained from all participants, and samples have been deidentified.

### PBMC-derived induced microglia-like cell (piMGLC) culture and seeding

piMGLCs were derived as previously described [27] with modifications. Frozen PBMCs (purchased from Vitrologic, Inc. for primary screen, or isolated from leukapheresis preparations obtained from Charles River, Lowell MA; Hemacare, Northridge CA, as described in Supplemental Methods, for secondary screen and transcriptomic analyses) were thawed at 37°C and transferred into RPMI-1640 (Sigma, #R8758) supplemented with 10% heat-inactivated FBS (Sigma, #12306C) and 1% Penicillin/Streptomycin (Life Technologies, #15140-122). After centrifugation at 300 *g* for 5 min (brake off), cells were resuspended, counted, and plated at ~500,000 cells/cm² in 6-well tissue culture plates (Corning, #353046) pre-coated with Geltrex (Gibco, #A1413202) for 1 hour. After 24 hours, media was replaced with RPMI-1640 containing 1% Penicillin/Streptomycin, 1% Glutamax (Life Technologies, #35050-061), 100 ng/ml IL-34 (Biolegend, #577904), and 10 ng/ml GM-CSF (PeproTech, #300-03). Cells were incubated for 8 days for transdifferentiation.

### Ribonucleotide protein (RNP) delivery of gRNAs

Guide RNAs (gRNAs) (Synthego library; Table S2) were resuspended to 25 μM. SpCas9 protein (IDT, #1081059) was diluted to 31 μM in Duplex buffer (IDT, #11-01-03-01) and combined with gRNAs at a 1:1.5 molar ratio (SpCas9:gRNA) to form ribonucleoprotein (RNP) complexes (~10.8 μM final concentration). Complexes were incubated for 10 min at room temperature, diluted to 7.2 µM, and kept on ice as 5 μl aliquots in 8-strip PCR tubes per 25,000 cells (per reaction).

piMGLC culture supernatant was collected, filtered through a 0.22 µm Steriflip-GP unit (EMD Millipore, #SCGP00525), and retained as Conditioned Medium. piMGLCs were washed with DPBS and detached using Accutase (Sigma, #A6964) for 5 min at 37 °C, centrifuged (300 *g*, 5 min), and resuspended in fresh medium.

For nucleofection, 25,000 cells (for assays) or 75,000 cells for Drug-seq were resuspended in 20 µl P3 buffer (Lonza P3 Nucleofection Kit, #V4SP-3096) and mixed with prepared RNPs. Cell-RNP suspensions were transferred into nucleofection cuvettes and nucleofected (Lonza 4-D nucleofector) using program EA-100. After a 10 min recovery at room temperature, cells were seeded into Geltrex-coated 96-well plates (Corning, #3904) with 180 µl Conditioned Medium (total 200 µl per well) and cultured for 6 days.

### Phagocytosis assays

Phagocytosis assays were conducted in 96-well plates (Corning, #3904) seeded with piMGLCs at a density 20,000 cells per well in 200 µl. Synaptosomes were thawed at room temperature and mixed 1:1 with 0.1 M sodium bicarbonate (pH 9), then labeled with pHrodo-Red dye (Invitrogen, #P36600, 6.67 µg/µl) at a protein:dye ratio of 2:1 for 1 hour. Labeled synaptosomes were washed by adding PBS (pH 7.4), pelleted at 12,000 rpm for 15 minutes, and resuspended in basal RPMI-1640 at 0.15 mg/ml. Synaptosomes were then sonicated in a Branson 1800 (Emerson, #M1800) at 40 kHz for 1 hour. Synaptosomes were added to each well at 3 µg/well using a multichannel pipette. After 3 hours, assays were terminated by fixation with 4% paraformaldehyde (Electron Microscopy Sciences, #15713S).

### RNA-seq data analysis

Raw paired-end FASTQ files were trimmed with Trim Galore v0.6.10 [33] and aligned to hg38 using STAR v2.7.11b [34]. The demultiplexed count matrix contained an average of 1.51 ± 0.39 million reads per sample. Differential expression was analyzed with DESeq2 [35]. For CRISPR-targeted genes, significance was defined as unadjusted *p* < 0.1, while for all other genes, FDR-adjusted padj < 0.1 was used, based on the rationale that expression of the perturbed gene is expected to change, reducing the likelihood of false positives relative to transcriptome-wide tests. Functional enrichment of DE genes with >10 DEGs per target was performed using clusterProfiler enrichGO [36]. Code is available at https://github.com/mgb-cqh/crispr30.

Additional details are in Supplementary Methods.

## Results

### Targeted functional genetic screening in human primary PBMC-derived reprogrammed microglia-like cells identifies genes that modulate synaptosome phagocytosis

To curate SCZ liability genes, we focused on genes that are both expressed in microglia [9, 37, 38] and found to be differentially expressed in SCZ in postmortem studies [7, 8, 39–41], particularly in the dorsolateral prefrontal cortex (DLPFC) [42]. We curated a set of ~200 genes from these datasets (Table S1) and selected those determined by gene ontology analysis (KEGG and GSEA) to be involved in phagocytosis, resulting in 30 genes for initial screening. (Fig. S1 and Table S1).

Due to the primary nature of the PBMCs, which cannot be passaged like cell lines engineered to express Cas9 protein [43–45], we adapted a modified protocol [46, 47] for CRISPR-mediated genome editing using Cas9/sgRNA ribonucleoprotein (RNPs) delivery of purified recombinant Cas9 complexed with sgRNAs, resulting in efficient manipulation of PBMC-induced microglial-like cells (piMGLCs) for use in screening SCZ-related genes.

**Fig. 1:**
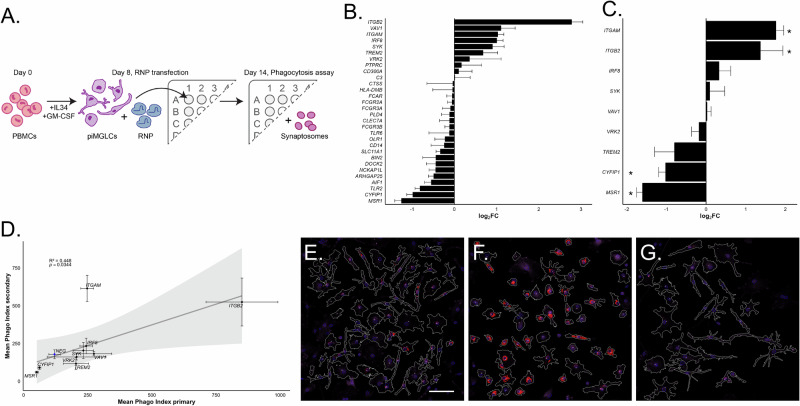
Image-based arrayed CRISPR screen for synaptosome phagocytosis modulators in piMGLCs. **A** Overview workflow for the derivation of piMGLCs, ribonucleoprotein (RNP) transfection, and assay. **b**BPhagocytosis primary screen of 30 curated SCZ-associated genes ranked by phagocytic index indicated as fold-change (FC) compared to the non-targeting gRNA negative control. (*n* = 3 replicate well mean values, # cells per well in Table S3, error bars indicate SEM). See Fig. S3 for image-field mean values (*n* = 111 image fields/3 wells) and test statistics. **C** Phagocytosis secondary screen of 9 SCZ-associated genes selected from the primary screen. Phagocytic index indicated as fold-change (FC) compared to non-targeting gRNA negative controls. (*n* = 3 replicate well mean cellular values, # cells per well in Table S4, error bars indicate SEM). Statistical significance was derived using a one-tailed Mann-Whitney U test (direction informed by primary screen results) for each gene target versus control, with Benjamini-Hochberg correction for multiple testing. Asterisks mark gene targets with adjusted *p*-value < 0.05. **D** Correlation of phagocytic indices between primary and secondary screens. Error bars indicate SEM, grey area indicates 95% confidence. Representative masked cell images used for quantification, white mask outline containing pHrodo stained synaptosomes (red) corresponding to **E** non-targeting gRNA negative control and gRNAs targeting **F** *ITGB2* and **G** *MSR1*. Scale bar = 100 μM.

Using this arrayed RNP-based CRISPR system with three sgRNAs per target (Table S2) per well in 96-well plate format, we performed gene targeting in piMGLCs (Fig. S2). Six days post sgRNA delivery, we performed phagocytosis screens (Fig. 1A) using pHrodo dye-labeled human iPSC-derived neuronal synaptosomes in a model of synaptic pruning [21, 26], followed by immunocytochemistry (ICC) to identify microglia by IBA1 + , and high-content confocal imaging to quantify phagocytic index (see Supplemental Methods). This primary screen revealed seven potential repressors of phagocytosis (i.e. gene targeting *increased* phagocytosis) compared to the non-targeting negative control sgRNAs (NEG) (Fig. 1B, Table S3). Conversely, the majority of genes reduced phagocytic activity upon gene targeting.

To determine independent reproducibility and confirm findings from the primary screen, secondary screening was conducted on nine selected target genes found to alter phagocytic function in the primary screen based on fold change effect size (top seven activators and top two repressors) and image-field based statistics in primary screening (Fig. S3) using redesigned, orthogonal sgRNAs (two per gene, Table S2) and additional negative controls, including an eGFP sgRNA (eGFP gene is not present in these cells) added to the non-targeting gRNA sequence (NTS) used in the primary screen. Consistent with primary results, *ITGB2* and *ITGAM* targeting increased phagocytosis while *MSR1* and *CYFIP1* targeting reduced phagocytosis (Fig. 1C, Table S4). Additionally, none of the targets reduced viability compared to non-targeting controls (Tables S3 and S4 and Fig. S4).

Correlation between primary and secondary screening results was observed for most treatments (R^2^ = 0.448, across genes, Fig. 1D), other than *ITGAM*. Excluding *ITGAM*, which by visual inspection was a marked outlier, primary and secondary results were highly correlated (R^2^ = 0.936 excluding *ITGAM*, Fig. S5), possibly reflecting different sgRNA editing efficiencies used between screens.

### Phenotypic screening identifies genes that modulate microglial morphology and activation state

We previously demonstrated that the morphometric parameters of solidity and eccentricity can be used to indicate morphological classes of microglia: ramified, ameboid, and intermediate bipolar/rod-shaped [27, 48]. Microglia reside in a surveillant state in the adult brain, characterized by a ramified morphology [49], but they alter their morphology with changes in functional state. This allows morphology to serve as a proxy for activation status, with ramified cells representing a surveillant state and ameboid cells indicating one of the multiple known microglial activation states [50].

**Fig. 2:**
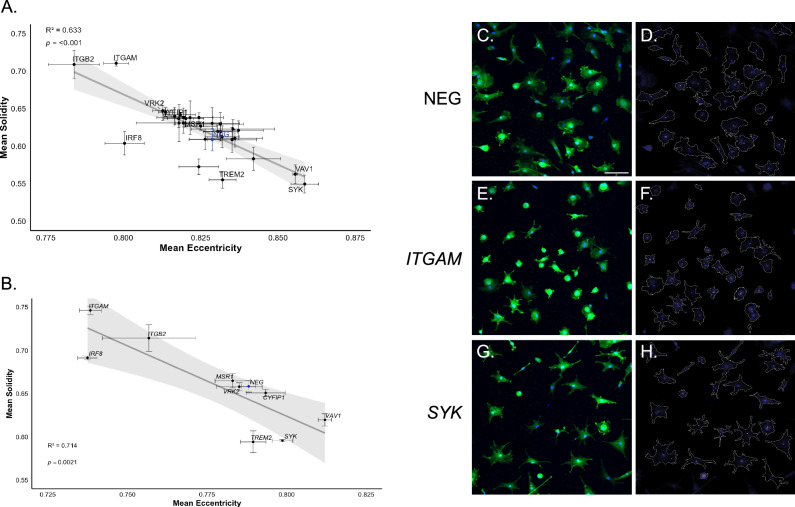
Phenotypic screening identifies genes that modulate piMGLC morphology. **A** Morphometric parameters, solidity versus eccentricity for 30 indicated CRISPR targeted genes in the primary screen. (*n* = 3 replicate well mean cellular values, # cells per well in Table S3). **B** Morphometric parameters: solidity versus eccentricity for 9 CRISPR targeted genes selected from primary screen. (*n* = 3 replicate well mean cellular values, # cells per well in Table S4). Error bars indicate SEM, grey area indicates 95% confidence. (**C**, **E**, **G**) Representative images (IBA1+ green, nuclei blue) and (**D**, **F**, **H**) corresponding masked cell outlines for quantification of indicated non-targeting gRNA negative control (NEG) and gRNAs targeting *ITGAM* and *SYK*. Scale bar = 100 μM.

To assess morphology after gene targeting, we used our arrayed image-based screening platform to simultaneously measure morphometric parameters and phagocytic function in both the primary and secondary screens (Fig. 2). We analyzed these piMGLCs using CellProfiler [51], extracting solidity and eccentricity values to infer microglial activation states by morphology.

In the primary screen, we observed pronounced changes in cellular morphometric parameters following targeting of several genes, including *ITGB2, ITGAM, SYK, VAV1, TREM2, IRF8*, and *VRK2* (Fig. 2A, Table S3). Targeting these genes prompted distinct morphological changes exhibiting shifts towards a ramified (high eccentricity, low solidity) or ameboid (low eccentricity, high solidity) morphology. For instance, targeting of *ITGB2* and *ITGAM*, both components of the complement receptor 3 (CR3) [52], exhibited increased solidity and reduced eccentricity relative to negative controls, consistent with a more ameboid morphology. Other gene targets, such as *VAV1* and *SYK* resulted in more ramified morphology, demonstrating varied effects on morphology across gene targets.

Morphometric analyses of the secondary screen indicate the morphological alterations observed in the primary screen were reproduced (Fig. 2B–H, Table S4) with many of the induced changes present in the absence of synaptosomes (Fig. S6, Table S5). We observed strong correlations between the two screening rounds for both solidity and eccentricity (R² = 0.82 and R^2^ = 0.708, respectively, Fig. S7), indicating reproducible morphology phenotypes upon gene-specific disruption across biological replicate screens.

**Fig. 3:**
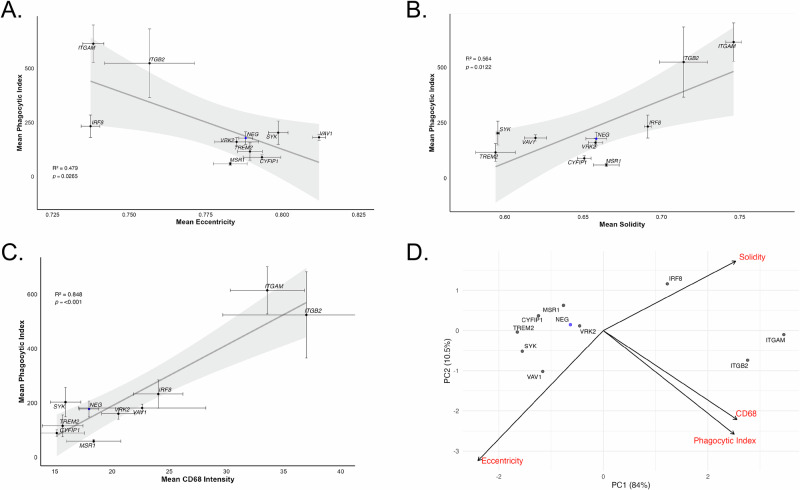
Functional and phenotypic analyses between targeted genotypes indicate correlations between phagocytic activity, morphology, and microglial activation marker expression. **A** Increased eccentricity results in lower phagocytosis while **B** increased solidity correlates with higher phagocytosis. **C** Correlation between phagocytic index and CD68 expression. (*n* = 3 replicate well mean cellular values, # cells per well in Table S4). Error bars indicate SEM, grey area indicates 95% confidence. **D** Multi-dimensionality reduction using principal component analysis (PCA) of solidity, eccentricity, phagocytosis, and CD68 marker expression enables discrimination between gene-targeted groups.

We found that morphology correlated with phagocytic index (Fig. 3A, B). Specifically, increased eccentricity was associated with lower phagocytosis (R^2^ = 0.479), while increased solidity was generally associated with higher phagocytosis (R^2^ = 0.564) across the genotypes examined. Overall, more ameboid, less ramified microglia tended to be more phagocytic.

To further explore relationships between targeted genotypes and functional states, we incorporated the expression level of the activation marker CD68 [53, 54] into our evaluations of microglial activation. CD68 expression levels were highly dispersed across genotypes (Fig. 3C), indicating specific gene targets lead to increased (e.g., *IRF8, ITGAM*, and *ITGB2*) or decreased (e.g., *TREM2, CYFIP1*, and *SYK*) activation compared to negative controls. Furthermore, CD68 expression positively correlated (R^2^ = 0.848) with phagocytic activity, suggesting coordinated changes between activation and phagocytic function.

We applied dimensionality reduction (Fig. 3D) using principal component analysis (PCA) of solidity, eccentricity, phagocytosis, and CD68 expression to understand the relationship among these phenotypes. These multidimensional phenotypic profiles separate functional microglia states that result from targeting genes. For example, *ITGAM* and *ITGB2* targets clustered upon PCA, exhibiting high activation and phagocytic capacity profiles, while *CYFIP1*, *TREM2*, and *MSR1* demonstrate high similarity due to lower activation and phagocytic function. This approach highlights that measures of microglia state cannot be captured by individual parameters. It further emphasizes the potential for high-throughput image-based assays to reveal unexpected or nuanced gene function relevant for prioritizing further disease modeling efforts, such as clonal gene knockout in human iPSC-derived microglia.

### Transcriptomic profiling reveals diverse gene targeting effects on microglial state

We next performed DRUG-seq [32, 55], a multiplexed RNA-sequencing approach optimized for high-throughput screening, to investigate the transcriptomic consequences of individual gene targeting in piMGLCs at steady state in the absence of synaptosomes.

**Fig. 4:**
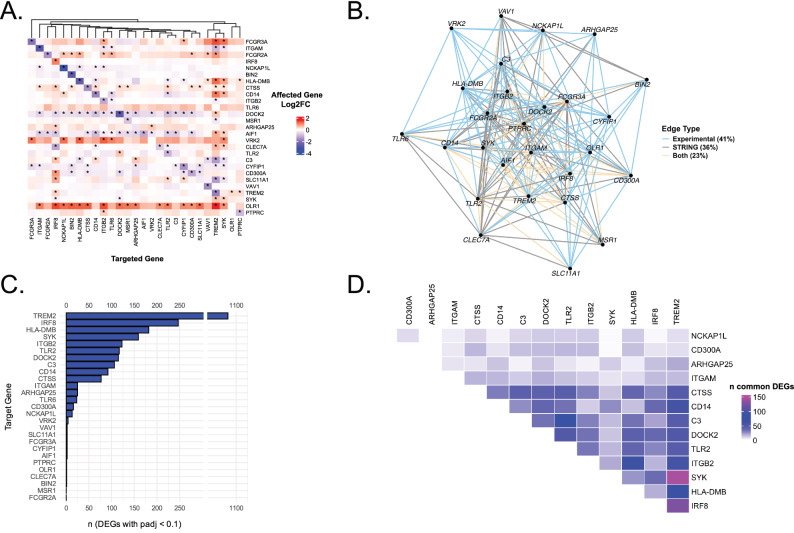
Differential Expression in Drug-Seq. **A** Pairwise effects of each target gene on expression of all other targeted genes. Colors show log2 fold change. Asterisks mark combination with *p*-value < 0.1. **B** Comparison with documented STRING-db pairwise gene interactions. Lines indicate (blue) current experimental only (41%), (grey) STRING-db only (36%), (yellow) both experimental and STRING-db (23%). **C** Number of DEGs (padj < 0.1) per target gene. Bar colors reflect the DEG status for that target gene (red = pvalue < 0.1 & log2FC > 0; blue = *p* value < 0.1 & log2FC < 0; black = otherwise). **D** Overlap of DEG in common between gene targets (minimum 5 DEG in common with at least one other gene target).

For each of the 30 targeted genes, we generated three biological replicate libraries and compared their expression profiles with non-targeting negative control (NTCs) samples processed in parallel. Three of the targeted genes (*PLD4*, *FCGR3B*, and *FCAR*) were minimally expressed in the piMGLCs (average coverage in controls <2 CPM, Fig. S8) and did not show an effect on phagocytosis upon targeting (Fig. 1B), likely due to their lack of intrinsic expression; thus were removed from further analyses. Of the remaining expressed genes, six did not result in significant changes in their own transcript levels: *ARHGAP25*, *SLC11A1*, *C3*, *TLR6*, *OLR1* and *VRK2* (raw *p*-value < 0.1, as shown by the diagonal in Fig. 4A). This may indicate that the selected gRNAs failed to cause nonsense-mediated decay (NMD) [56, 57] of their target transcripts, even though some of these resulted in modest changes in phagocytosis (*e.g. VRK2*, Fig. 1B), suggesting they may still alter protein function through mixed non-clonal functional mutation and/or out-of-frame indel-induced truncations. The remaining targets (21/30) resulted in significant alterations in their respective target gene transcript levels compared to NTCs, with genes such as *ITGAM*, *NCKAP1L*, *BIN2*, *HLA-DMB*, and *DOCK2* among the most reduced.

Interestingly, initial RNA sequencing indicated that targeting *IRF8* and *SYK* led to increased expression of their transcripts. However, further confirmation by qRT-PCR (Fig. S9) only supported this observation for *IRF8*, not *SYK*. Similarly, Western analysis of these gene targets indicated that the IRF8 protein level was slightly increased, while protein expression of SYK was markedly reduced. These results suggest possible alternative translation initiation (ATI) leading to increased transcript stability [57] in the former, and/or lack of nonsense-mediated transcript decay (NMD) in the latter [56, 57]. While this does not rule out the introduction of non-functional mutations in either of these proteins, it remains likely that both proteins were impacted, given our pronounced functional and phenotypic observations.

To examine potential regulatory relationships among the targeted genes, we cross-referenced expression of all 30 target genes across the DEG results from each perturbation, generating a pairwise differential expression matrix (Fig. 4A). The targeting of several genes (e.g., *TREM2*, *SYK* and *IRF8*) triggered broad transcriptomic changes in other targets, indicated by significant log₂ fold changes (FC). Alternatively, expression differences in some affected genes (e.g., *DOCK2*, *AIF1*, and *OLR1*) were induced by many targeted genes, suggesting downstream regulation by other targeted genes. Many of these pairwise comparisons (23%) corresponded to documented interactions in the STRING database [58] (Fig. 4B). In contrast, 41% of the identified relationships have not been reported in STRING and may represent microglia-specific regulatory networks.

Further transcriptomic analysis identified genes that exert the most profound impacts on the transcriptome following CRISPR targeting. Several gene targets are notable for driving the largest numbers of significantly differentially expressed genes (DEGs) (Fig. 4C, Table S7), such as *TREM2*, *IRF8*, *HLA-DMB*, and *SYK*, representing loci whose perturbation results in widespread transcriptional reprogramming. Notably, targeting *ITGB2* resulted in approximately 5-fold more DEGs than *ITGAM*, despite both encoding subunits of the CR3 receptor [52], suggesting non-overlapping functions.

Pairwise analyses that show the most significant overlap in DEG profiles (Fig. 4D) highlight potential candidate interactions or shared pathways. The most notable overlaps, indicated by the largest intersecting DEGs (e.g., *TREM2* and *SYK* are genes within the same phagocytic and activation pathways [59]) emphasize gene pairs whose parallel activity may contribute to pathway outputs or whose combined loss uncovers new regulatory dependencies.

The biological implications of these findings suggest gene targets that yield the highest number of DEGs may serve as regulatory hubs or critical nodes within microglial functional pathways, and that gene pairs with maximum DEGs overlap identify possible points of redundancy in gene networks. Identifying such genes and pairs informs prioritization for mechanistic follow-up, functional assays, or therapeutic targeting.

### Functional programs induced by targeted genes

**Fig. 5:**
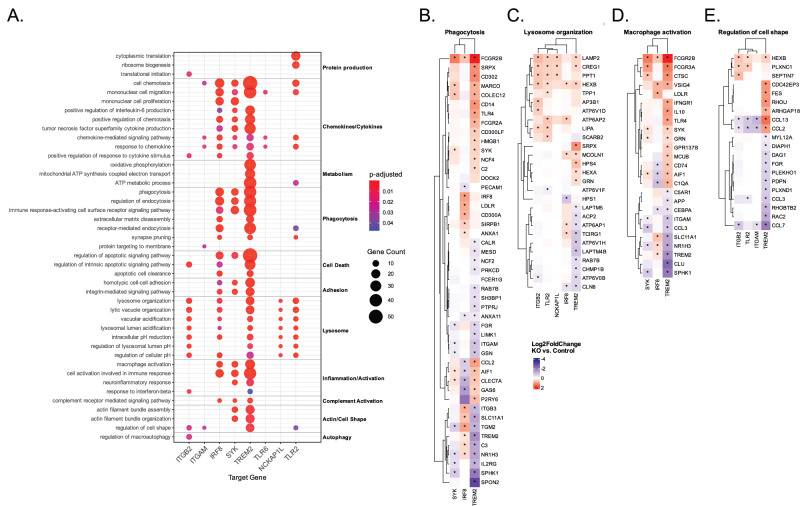
Transcriptomic analysis of targeted genes. **A** Select GO Biological Process enrichment for the target gene DEG. Dot color encodes adjusted p-value. Dot size encodes term gene count. Gene-level differential expression heatmaps for select GO Biological Process terms enriched in the DEGs of target genes **B** phagocytosis, **C** lysosome organization, **D** macrophage activation, and **E** regulation of cell shape. Log2FC = log₂ fold change (Targets vs Control); red indicates up-regulation and blue indicates down-regulation. Asterisks mark genes with |log₂FC|> 0.2 and adjusted *p*-value (padj) < 0.1. Only treatments/genes with available data are displayed.

GO Biological Process enrichment highlighted functional themes relevant to microglial biology. We curated the enriched processes into a set of broad functional categories that are biologically relevant and represented across targets, including chemokine/cytokine signaling, lysosomal pathways, phagocytosis, cell death, cell adhesion, actin cytoskeletal remodeling, metabolism, protein production, complement activation, and autophagy (Fig. 5A, Table S8).

Targeting *TREM2* and *SYK* affected processes central to microglial function, including phagocytosis, lysosome organization and acidification, cell activation, cytokine/chemokine signaling, and actin cytoskeletal remodeling [59–61], supported by our functional and phenotypic data. *IRF8* and *TREM2* targeting affected the expression of numerous other targeted genes (Fig. 4D) and a broad set of GO processes, confirming central roles in microglial regulation [62]. *ITGB2* and *ITGAM*, despite encoding subunits of the CR3 receptor (CD11b and CD18, respectively) [14, 63], showed divergent enrichment patterns due to *ITGB2*’s broader transcriptomic impact (Fig. 4C).

Beyond these major regulators, targeting *TLR2* impacted protein production and lysosomal acidification processes, indicating effects on fundamental cellular processes such as translation and degradation. *NCKAP1L* targeting showed more specialized enrichment, particularly in lysosomal acidification and actin cytoskeletal organization, consistent with its known role in the WAVE complex and phagocytosis [64, 65].

We compared gene targets by examining the gene-level changes in four strongly enriched processes (Fig. 5B–E). First, phagocytosis was a strongly enriched process across *SYK*, *IRF8*, and *TREM2* targets (Fig. 5A). TREM2 affected the largest number of genes (Fig. 5B) in this category [39], including upregulation of canonical phagocytic receptors and effectors such as *FCGR2A/B*, *CD14*, *MARCO*, *CD302*, *CLEC7A*, as well as downregulation of *ITGAM* and *C3*. IRF8 targeting influenced a partially overlapping set of 18 genes, but with predominantly opposite directionality, aside from consistent upregulation of FCGR2B and consistent downregulation of ANXA11. Targeting *SYK* affected 13 phagocytosis genes, including *FGR*, *MARCO*, *CLEC7A*, and *ITGAM*, and the direction of expression changes was broadly concordant with those observed in *TREM2*.

Lysosomal organization was also affected across multiple targets, including *ITGB2*, *TLR2*, *NCKAP1L*, and *TREM2*, highlighting disruption of phagolysosomal processes (Fig. 5C). Several lysosomal genes, including *LAMP2*, *CREG1*, and *HEXB*, were consistently upregulated across multiple targets, potentially reflecting lysosomal stress. In contrast, a broader collapse of lysosomal organization is represented by the following: *ATP6V0B* was downregulated in *ITGB2* and *TREM2* targets which indicates reduced acidification [66], *CLN8* was reduced in *TREM2* and *IRF8* targets, suggesting deficits in trafficking [67], and targeting *TREM2* exhibited the most extensive downregulation of lysosomal genes, including *CHMP1B*, *ACP2*, and multiple *LAPTM* family members.

Macrophage activation pathways were also prominently enriched across *TREM2, IRF8*, and *SYK* (Fig. 5D), with mixed overall effects on activation. Changes were generally modest upon targeting *IRF8*, with classical activation markers such as *C1QA*, *AIF1*, and *CD74* reduced, while homeostatic or anti-inflammatory genes, including *VSIG4* and *LDLR*, were increased. Targeting *SYK* and *TREM2* resulted in highly concordant transcriptional profiles and included strong upregulation of Fc receptor and antigen-processing genes such as *FCGR2B*, *FCGR3A*, *CTSC*, and *CD74*, as well as downregulation of inflammatory and metabolic regulators, including *SPHK1*, *NR1H3*, *CCL3*, and *CLU*. This pattern suggests that *SYK* and *TREM2* disruption elicits a shared transcriptional program characterized by reduced proinflammatory activation and enhanced antigen-presenting functions, despite decreased phagocytic activity in functional assays.

Finally, regulation of cell shape was enriched across several targets (Fig. 5E), reflecting both signaling-mediated and cytoskeletal remodeling processes. *TREM2* had the broadest impact, altering both upstream signaling components and downstream effectors, including small GTPase regulators (*RHOU, RAC2, CDC42EP3, ARHGAP18*) and cytoskeletal modulators (*DIAPH1, MYL12A*). In contrast, *ITGB2, ITGAM*, and *TLR2* primarily influenced chemokine expression (e.g. *CCL13*, *CCL2* and *CCL7*).

## Discussion

In this study, we investigated the functional roles of schizophrenia (SCZ)-associated genes in a human microglia-like cellular model using an arrayed CRISPR screen coupled with high-content imaging and transcriptomic profiling. Our results identify a subset of microglial-expressed genes that regulate phagocytosis, morphology, and activation states. These findings advance our understanding of microglial involvement in SCZ and demonstrate the utility of integrated functional genomics to determine disease-relevant cellular mechanisms.

The genes prioritized for CRISPR screening were curated from SCZ postmortem transcriptomic datasets [7] and further enriched by microglial expression [8, 9] and phagocytic genetic ontogeny (DAVID [68, 69] and GSEA [70, 71]). This approach enabled the functional validation of these candidates within a human microglial context, connecting large-scale association studies with mechanistic insight.

To address the limitations in arrayed CRISPR screening that can introduce confounding factors, including variable well-to-well editing efficiencies from RNP nucleofection, potential variable expression of target genes, and potential off-target effects, we designed orthogonal sgRNAs for secondary validation of selected targets in addition to incorporating additional negative controls (e.g., non-targeting gRNAs and eGFP, the latter not present in these cells) to compare with for well-level image data normalization. This method ultimately presents a relevant, efficient primary cell screening method that can be used to identify and characterize the functional outcome of gene disruption to design subsequent genetic studies using clonal iPSC-derived microglia and/or 3D culture systems for greater fidelity and resolution of cell-autonomous versus intercellular effects.

Among the most robust impacts on phagocytosis were increased activity following targeting of *ITGAM* and *ITGB2* and reduced activity in *MSR1* and *CYFIP1*. Many genes that demonstrated impacts on phagocytic uptake of synaptosomes are components of known pathways implicated in synaptic pruning, a process hypothesized to be dysregulated in SCZ [72–75]. The altered phagocytosis observed upon their targeting supports this model and suggests that deficits in microglial engulfment may contribute to the synaptic dysfunction observed in patients.

Our high-throughput multiplexed transcriptomic analyses provided a transcriptome-wide view of CRISPR targeting-induced changes. It revealed substantial heterogeneity among genes, both in the magnitude of transcriptional impact and in the pathways affected. Building on the analysis of interacting gene pairs, these pathway-level data reveal how individual targets give rise to convergent transcriptional programs. While some targets, such as TREM2, SYK, and IRF8, elicited broad transcriptional responses affecting multiple cellular programs, including phagocytosis, chemokine signaling, and lysosomal processes, other genes, such as ITGB2, ITGAM, NCKAP1L, and TLR2, showed more focused process enrichment, often linked to core effector functions such as actin remodeling, lysosomal acidification, or protein production.

Phenotypic effects did not consistently parallel transcriptomic breadth, as some genes produced broad transcriptional changes but modest phenotypic shifts, such as *IRF8* and *TREM2*, while others showed strong functional responses and relatively focused transcriptomic signatures, such as ITGB2 and ITGAM. SYK uniquely exhibited strong transcriptional and phenotypic effects, consistent with its role as a central signaling hub. This discordance highlights that different genes can influence microglial function through distinct regulatory modes, some acting via specific effector pathways that directly impact cellular behavior, and others through broader transcriptional networks that modulate microglial states in more complex ways. These insights point to distinct regulatory circuits disrupted by each gene and suggest potential points of convergence or synergy.

The integration of phenotypic and transcriptomic datasets supports a model in which subsets of SCZ-associated genes control microglial function through distinct mechanisms, some reducing phagocytic capacity directly through receptor or lysosome function, (e.g., *ITGB2*, *IRF8*, *TREM2*, *NCKAP1L* and *TLR2*), others also altering cellular activation or survival pathways (e.g., *IRF8*, *TREM2* and *SYK*) and/or through direct transcriptional regulation via transcription factors (e.g., *IRF8*). For instance, targeting *IRF8* led to increased expression of pro-inflammatory transcripts, suggesting an activation response rather than a direct phagocytic defect, thus highlighting the importance of multiplexed phenotyping strategies when interpreting the impact of gene perturbations in complex cellular systems. Other genes, such as *VRK2*, that resulted in functional and phenotypic disruptions upon targeting, with less understood connections to these other SCZ-implicated genes, warrant further investigation.

Our results support a model in which SCZ-associated genes modulate key microglial functions, including phagocytosis and immune activation, through distinct molecular mechanisms. More broadly, these findings present a scalable platform for functional genomics in microglia, enabling disease modeling and therapeutic discovery applicable to SCZ, and neurodevelopmental or neurodegenerative disorders. The identification of regulatory hubs and redundant gene networks provides key targets for further mechanistic follow-up and potential intervention strategies.

## Funding

This work was supported by the National Institute of Mental Health (NIMH) R01MH120227 (Perlis) and R01MH131687 (Sheridan and Perlis). The next-generation sequencing was performed in Tufts University Core Facility Genomic Core, which received funding support from NIH (S10 OD 032203) for its Illumina NovaSeq sequencer.

## Supplementary information

Supplementary Materials and Methods

Supplementary Data Table S1

Supplementary Data Table S2

Supplementary Data Table S3

Supplementary Data Table S4

Supplemental Data Table S5

Supplemental Data Table S6

Supplemental Data Table S7

Supplemental Data Table S8

## Data Availability

Gene expression data will be made available for download from the NCBI Gene Expression Omnibus (https://www.ncbi.nlm.nih.gov/geo/query/acc.cgi?acc=GSE319209) upon publication. Additional data that support the findings of this study will be available from the corresponding authors upon reasonable request.
